# Effect of herbivore load on VOC-mediated plant communication in potato

**DOI:** 10.1007/s00425-023-04075-6

**Published:** 2023-01-23

**Authors:** Carla Vázquez-González, Violeta Quiroga, Lucía Martín-Cacheda, Sergio Rasmann, Gregory Röder, Luis Abdala-Roberts, Xoaquín Moreira

**Affiliations:** 1 Department of Ecology and Evolutionary Biology, University of California-Irvine, Irvine, CA 92697 USA; 2 Misión Biológica de Galicia (MBG-CSIC), Apartado de Correos 28, 36080 Pontevedra, Galicia Spain; 3 Institute of Biology, University of Neuchâtel, Rue Emile-Argand 11, 2000 Neuchâtel, Switzerland; 4 Departamento de Ecología Tropical, Campus de Ciencias Biológicas y Agropecuarias, Universidad Autónoma de Yucatán, Apartado Postal 4-116, Itzimná, 97000 Mérida, Yucatán Mexico

**Keywords:** Herbivore density, Induced resistance, Plant–plant signalling, *Solanum tuberosum*, *Spodoptera exigua*, Volatile organic compounds

## Abstract

**Main conclusion:**

**VOC emissions increased with herbivore load, but this did not result in concomitant increases in resistance in neighbouring plants, suggesting that communication occurred independently of herbivore load in emitter plants.**

**Abstract:**

Herbivore-damaged plants emit volatile organic compounds (VOCs) that can alert neighbours and boost their resistance. While VOC-mediated plant communication has been shown to be herbivore-specific, we know little about its contingency on variation in herbivore load. To address this knowledge gap, we tested herbivore load effects on VOC-mediated communication between potato plants (*Solanum tuberosum*) using the generalist herbivore *Spodoptera exigua*. First, we tested whether herbivore load (three levels: undamaged control, low, and high load) affected total VOC emissions and composition. Second, we matched emitter and receiver plants and subjected emitters to the same herbivore load treatments. Finally, we performed a bioassay with *S. exigua* on receivers to test for induced resistance due to VOC-mediated communication. We found that herbivory significantly increased total VOC emissions relative to control plants, and that such increase was greater under high herbivore load. In contrast, we found no detectable effect of herbivory, regardless of the load, on VOC composition. The communication experiment showed that VOCs released by herbivore-induced emitters boosted resistance in receivers (i.e., lower leaf damage than receivers exposed to VOCs released by control emitters), but the magnitude of such effect was similar for both levels of emitter herbivore load. These findings suggest that changes in VOCs due to variation in herbivore load do not modify the outcomes of plant communication.

**Supplementary Information:**

The online version contains supplementary material available at 10.1007/s00425-023-04075-6.

## Introduction

Plant communication involves the emission of complex blends of volatile organic compounds (‘VOCs’ hereafter) by herbivore-induced plants (‘emitters’) that are perceived by undamaged conspecific or heterospecific neighbours (‘receivers’) (Heil and Karban 2010; Karban et al. 2014a). Perception of such chemical cues can lead to either priming (i.e., activation) or full induction of defences in receiver plants, ultimately resulting in increased resistance against herbivores (Karban 2015).

Recent advances suggest that the occurrence and strength of herbivore-induced plant communication is highly specific (Moreira and Abdala-Roberts 2019) and is contingent on factors such as herbivore identity or plant genotype (Karban et al. 2013, 2014b, 2016; Moreira et al. 2018). One ubiquitous factor potentially affecting communication is the number of herbivores on a plant (“herbivore load” hereafter) as this relates to the amount of plant damage. Variation in herbivore load often results in dose-dependent plant induced responses, whereby defence induction increases with the amount of damage (Karban and Baldwin 1997; Karban 2011). Accordingly, studies have shown that differences in herbivore load and subsequent damage results in concomitant changes in VOC emissions and composition (Shiojiri et al. 2010; Girling et al. 2011; Pinto-Zevallos et al. 2018). It is therefore reasonable to expect that herbivore load-dependent VOC-induced responses impact plant signalling, but this possibility remains untested.

In this study, we investigated the effect of herbivore load on VOC emissions and VOC-mediated communication between potato (*Solanum tuberosum*) plants using larvae of *Spodoptera exigua*, a generalist leaf-chewing insect. Specifically, we asked whether variation in herbivore load influenced the total amount and composition of VOC emissions, and whether this, in turn,  influenced plant communication. We first performed an experiment to test for changes in total VOC amount and composition in plants subjected to one of the following treatments: undamaged control, low herbivore load, and high herbivore load. Next, in a separate experiment, we paired potato plants (i.e., emitter and receiver) and subjected emitter plants to the same above herbivore load treatments. Finally, we conducted a bioassay on receiver plants to test for herbivore load effects on the amount of leaf area consumed by *S. exigua* (i.e., induced resistance). By doing so, the present study advances our understanding of herbivore load-dependent plant communication via VOCs, a potentially widespread source of variability in chemically mediated plant-herbivore interactions.

## Materials and methods

### Experimental design

First, we performed a greenhouse experiment to test for effects of herbivore load on the emission of VOCs in potato plants (experiment 1). We sowed 60 tubers (Agricola Roberto, Pontevedra, Spain) from four *S. tuberosum* cultivars (15 tubers per cultivar: Agria, Baraka, Desiree, and Monalisa) in individual 4 L pots containing potting soil. We grew plants in a glasshouse under controlled light (minimum 10 h per day, photosynthetically active radiation = 725 ± 19 μmol m^−2^ s^−1^) and temperature (10 °C night, 25 °C day), and watered them twice a weak. We included different potato cultivars to increase variability, since the strength of plant signalling and VOC emissions have been shown to depend on the genotype identity of emitter and receiver plants (Kariyat et al. 2012; Karban et al. 2014b, 2016). One month after germination, we randomly assigned 20 plants to each of the following herbivore load treatments: (1) undamaged control (no damage), (2) subjected to two third-instar larvae of *S. exigua* on one fully expanded leaf (“low load”), or (3) subjected to four third-instar larvae of *S. exigua,* two on each of two fully expanded leaves of similar size and position within the plant (“high load”). Larvae were obtained from eggs and fed on a wheat germ-based artificial diet prior to the experiment. Cultivars were equally distributed among treatments. We covered leaves with a netting nylon bag to prevent larval dispersal. For control plants, we also covered one leaf with a netting nylon bag but without adding larvae to control for bagging effects. After 4 days of herbivore feeding, we removed larvae and collected VOCs on charcoal filters and analysed samples with gas chromatography coupled to mass spectrometry (GC–MS), as described in Rasmann et al. (2011). All VOCs were primarily identified using the NIST database before being confirmed using either commercially available standards or comparing their Kováts retention indices, calculated relative to the retention times of a series of n-alkanes (C_8_–C_20_, Sigma-Aldrich, Merck KGaA, Darmstadt, Germany) analysed under the same chromatographic conditions, with those reported in the literature (Table S1). We quantified emission of individual VOCs using normalized peak areas and expressed it as nanograms per hour (ng h^−1^). We obtained normalized peak area of each individual compound by dividing their integrated peak areas by the integrated peak area of the internal standard (tetraline, CAS#119-64-2) (Abdala-Roberts et al. 2022), to standardize for variations in the sample volume during the elution process. Reported values for individual VOCs should thus be considered as tetralin-equivalent nanograms of compound released by each plant per hour. The total emission of VOCs was then calculated as the sum of all individual VOCs.

Second, we performed another glasshouse experiment under the same environmental conditions to test whether effects of herbivore load on VOC induction lead to changes in the strength of plant communication (experiment 2). We sowed 120 tubers (Agricola Roberto, Pontevedra, Spain) from five *S. tuberosum* cultivars (24 tubers per cultivar: Agria, Baraka, Desiree, Kennebec, and Monalisa) as described above. One month after germination, we paired the plants in 37.5 × 37.5 × 96.5 cm plastic cages (*n* = 60 replicates) to avoid VOCs cross-communication among replicate pairs. Within each cage, one plant served as emitter (average height ± SE = 25.05 ± 0.89 cm) and the other acted as receiver (23.92 ± 0.94 cm). Emitter and receiver plants were placed ca. 20 cm apart to avoid physical contact but still close enough to assure VOC-mediated signalling between neighbouring plants as shown by other studies in natural settings (Karban et al. 2006; Heil and Adame-Álvarez 2010). One month after germination, we randomly assigned emitter plants within each cage to one of the above treatment levels, namely no damage (control), low, or high herbivore load (*n* = 20 cages per level). Emitter and receiver plants were always of the same variety and varieties were equally distributed across treatments (four replicates per cultivar per treatment). After 4 days of herbivore feeding, we removed emitter plants from the cages and set up a resistance bioassay on receivers. Briefly, we placed one third-instar *S. exigua* larva on each of two leaves on all receiver plants and left them feed for 3 days. We measured the percentage of leaf area removed (‘leaf damage’ hereafter) in both emitters and receivers using the mobile application BioLeaf—Foliar Analysis™ (Brandoli Machado et al. 2016). Average leaf damage in emitters was 12.17% and did not differ among treatments (*F* = 0.45, *P* = 0.506) or replicated leaves within the high herbivore load treatment (*F* = 1.59, *P* = 0.225). Because leaves were of similar size, by adding an extra pair of caterpillars in an additional leaf in the high herbivore load treatment we assumed double the amount of damage in these plants.

### Statistical analyses

First, using data from experiment 1 (effects on VOCs’ emissions), we ran general linear mixed models to test for the effect of herbivore load treatment (fixed factor with three levels: control, low, and high load) on the total amount of VOCs emitted as well as on individual compounds. We included plant height as a covariate and potato variety as random factor in the models and log-transformed total VOC emissions to achieve normality of model residuals. If the treatment effect was significant, we conducted post hoc Tukey’s HSD comparisons to test for pairwise differences between groups. In addition, we ran a Permutational Multivariate Analysis of variance (PERMANOVA) based on 10,000 permutations to test the effects of herbivore load on VOC composition using Bray–Curtis dissimilarity matrices based on abundances of each individual compound while controlling for genetic variation potentially affecting VOCs’ emissions. We then conducted a Principal Coordinate Analysis (PCoA) based also on Bray–Curtis pairwise dissimilarities to visualize changes in VOC composition (Moreira et al. 2021).

Second, using data from experiment 2 (communication effects), we ran a general linear mixed model to test for the effect of emitter herbivore load (fixed factor) on leaf damage in receiver plants. We included the individual plant and potato variety as random factors to account for non-independence between leaves sampled on each plant and to control for plant genetic relatedness, respectively. We also included plant height as a covariate to control for differences in size potentially affecting induced defences. We square-root-transformed mean leaf damage to achieve normality of model residuals. If the treatment effect was significant, we conducted post hoc Tukey’s HSD comparisons to test for pairwise differences between groups.

We ran all statistical analyses in R software version 4.2.1 (R Core Team 2013). We ran linear mixed models using the *lmer* function from the *lmerTest* package (Kuznetsova et al. 2017). We obtained least-square means and standard errors from general linear mixed models and ran post hoc mean contrast analysis using the *lsmeans* function from the *lsmeans* package (Lenth 2016). We ran PERMANOVA analysis and ordination methods using the *adonis* and *capscale* functions, respectively, both from the vegan package (Oksanen et al. 2010).

## Results

**Fig. 1:**
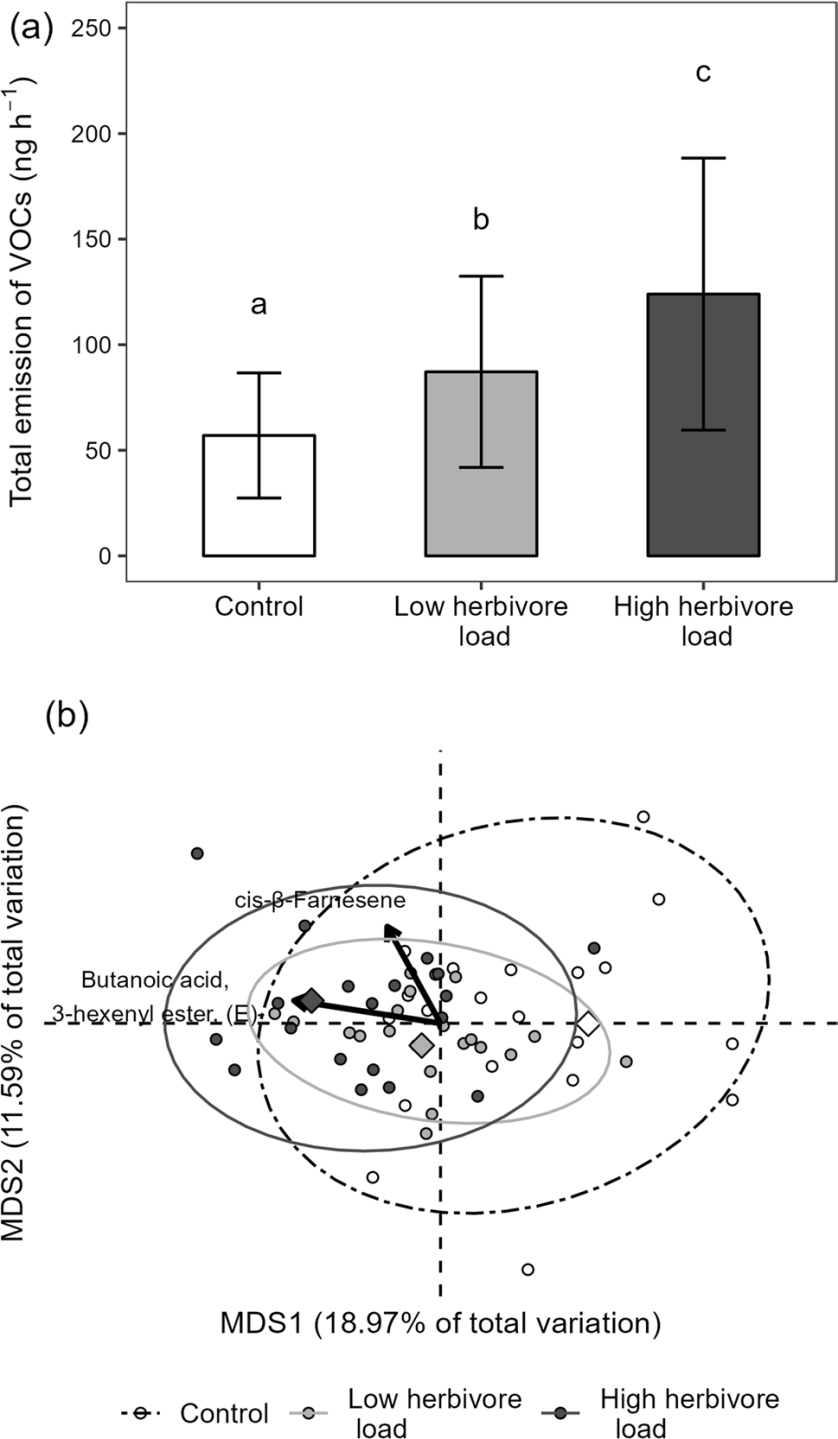
**a** Total emission of volatile organic compounds (VOCs; measured as normalized peak areas in nanograms per hour) in response to herbivore load treatments in emitter plants (namely: undamaged control, low, and high load of *Spodoptera exigua* in emitter potato (*Solanum tuberosum*) plants. Bars are back-transformed least-square means ± SE obtained from a general linear mixed model (*n* = 20); see Materials and methods. Different letters indicate significant differences (*P* < 0.05) between least-square means. **b** Unconstrained ordination (PCoA) showing the effect of the herbivore load treatment on the composition of VOCs for plants from a second experiment aimed to test for effects on emissions. Biplot arrows represent linear associations for the first two most influential VOCs (i.e., those having the strongest associations with the first two ordination axes). Diamonds represent the centroids for each emitter herbivory treatment and associated 95% ellipses

We identified a total of 31 VOCs emitted by *S. tuberosum* plants (Table S1). The herbivore load treatment significantly affected the total emission of VOCs (Table 1), whereby both low and high load plants significantly increased total VOC emissions relative to controls (52.9% and 117.5%, respectively), and high load plants showed a significant 42.23% increase in VOC emission compared to low load plants (Fig. 1a). Analyses by compound indicated that herbivory significantly increased the emission of 12 VOCs, with only one of them, the butanoic acid, 3-hexenyl ester, (E), showing a significant increased emission between the low and high load treatments (Table S1). However, the PERMANOVA analysis indicated no significant effect of the herbivore load treatment on VOC composition (Table 1; Fig. 1b).

**Table 1:** Effect of herbivore load treatment in emitter plants (undamaged control, low load, and high load of *Spodoptera exigua*) on the total emission (log-transformed data) and composition of volatile organic compounds (VOCs) released by emitter potato (*Solanum tuberosum*) plants, and on the percentage leaf area damaged by *S. exigua* on receiver plants exposed to emitter VOCs (square root-transformed data)

	DF	*F*/Pseudo-F	*P*
Total emission of VOCs	2, 53	16.63	** < 0.001**
Composition of VOCs	2, 59	1.67	0.089
Leaf damage	2, 53	9.7	** < 0.001**

*F* values/Pseudo-F for each factor, degrees of freedom (DF), and associated *P* values obtained from general linear mixed models in the case of total VOC emissions and leaf damage, and from PERMANOVA in the case of VOC composition are reported. Significant *P* values (*P* < 0.05) are highlighted in bold

**Fig. 2:**
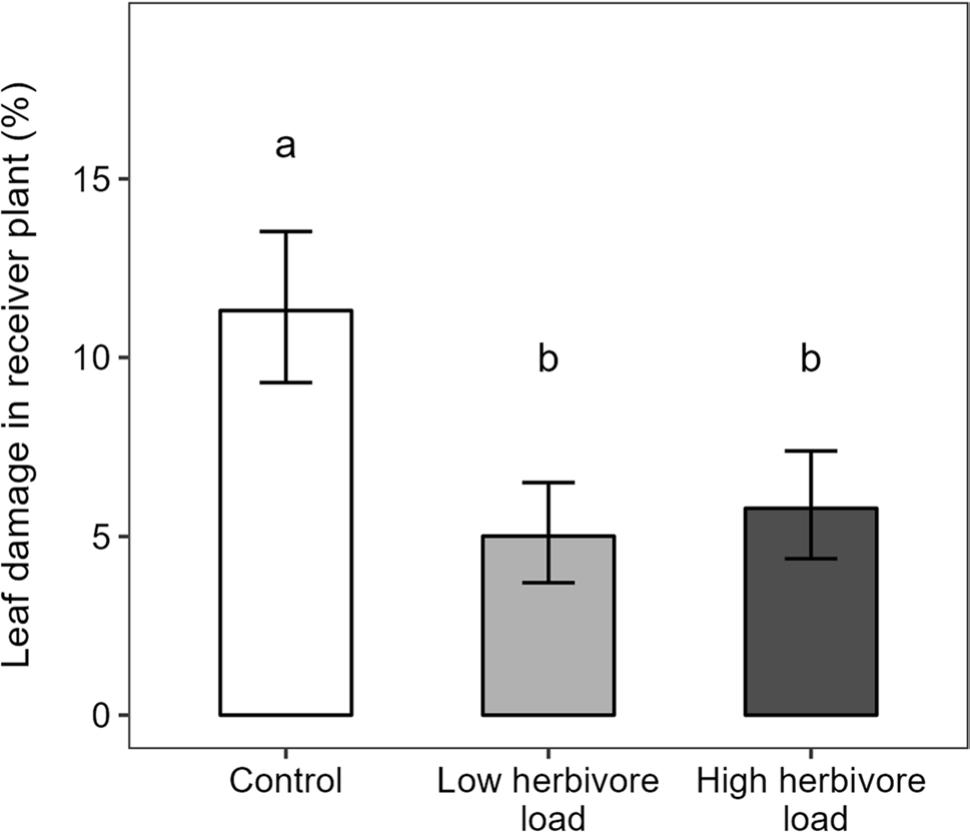
Percentage of leaf area consumed by *Spodoptera exigua* on receiver potato (*Solanum tuberosum*) plants in response to herbivore load treatments in emitter plants (control, low and high load of *Spodoptera exigua*). Bars are back-transformed least-square means ± SE obtained from a general linear mixed model (*n* = 20). Different letters indicate significant differences (*P* < 0.05) between least-square means

Results from the receiver bioassay indicated that the emitter herbivore load treatment significantly affected leaf damage on receiver plants (Table 1). Specifically, we found that low and high load plants both suffered a significantly lower amount of leaf damage relative to controls (55.7% and 48.8% decrease, respectively), but did not differ themselves (Fig. 2).

## Discussion

Our results indicated that herbivory by *S. exigua* increased total VOC emissions as well as the emission of several individual compounds. Specifically, total emission of VOCs increased with herbivore load, being the lowest in control plants, intermediate in low load plants, and the highest in high load plants. Similar load-dependent effects of herbivory on VOC emissions have been reported for other plant species, such as *Brassica oleracea* (Shiojiri et al. 2010; Girling et al. 2011) and *Manihot esculenta* (Pinto-Zevallos et al. 2018). Contrarily, we found no detectable compositional changes in VOCs due to herbivore load, whereas previous work with *Brassica oleracea* reported compositional changes in volatile profiles with increasing levels of herbivore damage (Shiojiri et al. 2010; Girling et al. 2011). It should be noted, however, that analyses by individual compounds showed that VOCs were differentially induced (i.e., 12 of 31 compounds were induced) suggestive of some degree of compositional changes in VOC blends (albeit mostly independent of load) due to herbivory.

Consistent with findings in other crops (Heil and Karban 2010), we found evidence of VOC-mediated communication in response to herbivory. Specifically, receiver potato plants exposed to VOCs from herbivore-induced emitters had lower leaf damage by *S. exigua* compared to receivers exposed to VOCs from control emitters. However, contrary to expectations, positive effects of herbivore load on total VOC emissions did not lead to corresponding increases in induced resistance, given by a lack of statistical difference in leaf damage between receivers exposed to emitters with low vs. high herbivore load. It is possible that the difference in herbivore loads and resulting total VOC induction was not large enough to drive concomitant responses in receiver plants exposed to low vs. high load emitters. Another non-exclusive explanation is that there are physiological constraints and allocation costs to defence induction that limit receiver responses to incoming VOCs beyond certain thresholds, such that receivers are not able to exhibit further increases in induced defences (and resulting resistance) concomitant to the magnitude of VOCs changes. Accordingly, and in agreement with our results, Girón-Calva et al. (2012) found that different concentrations of two VOCs caused similar levels of resistance to pathogenic bacteria in *Phaseolus lunatus* after exposure time to such VOCs exceeded 24 h. Finally, it is also possible that communication effects respond to induced emissions of specific, biologically meaningful, VOCs. Further studies testing more contrasting herbivore load levels and a greater load range, combined with detailed experimental tests of individual VOCs and blends, are needed to test load-dependent induced changes in VOCs and resulting variation in plant communication.

### *Author contribution statement*

Formulated the idea of the manuscript and designed the experiment: XM, CVG, and LAR. Performed the experiment: CVG, VQ, XM, and LMC. Performed the chemical analyses: GR, SR, and LMC. Contributed reagents/materials/analysis tools: XM, SR, and GR. Analysed the data: CVG. Wrote the first draft of the manuscript: CVG. Contributed critically to the writing: XM, LAR, SR, and GR. All authors gave final approval for publication and agreed to be held accountable for the work performed therein.

## Supplementary Information

Below is the link to the electronic supplementary material.

Supplementary file1 (DOCX 21 KB)

Supplementary file2 (TXT 15 KB)

Supplementary file3 (TXT 4 KB)

Supplementary file4 (TXT 1 KB)

## Data Availability

All data generated or analysed during this study are included in this article [and its supplementary information files].

## Abbreviation

VOC: Volatile organic compound
