# Correction: Effect of herbivore load on VOC‑mediated plant communication in potato

**DOI:** 10.1007/s00425-024-04546-4

**Published:** 2024-11-15

**Authors:** Carla Vázquez-González, Violeta Quiroga, Lucía Martín-Cacheda, Sergio Rasmann, Gregory Röder, Luis Abdala-Roberts, Xoaquín Moreira

**Affiliations:** 1 Department of Ecology and Evolutionary Biology, University of California-Irvine, Irvine, CA 92697 USA; 2 Misión Biológica de Galicia (MBG-CSIC), Apartado de Correos 28, 36080 Pontevedra, Galicia Spain; 3 Institute of Biology, University of Neuchâtel, Rue Emile-Argand 11, 2000 Neuchâtel, Switzerland; 4 Departamento de Ecología Tropical, Campus de Ciencias Biológicas y Agropecuarias, Universidad Autónoma de Yucatán, Apartado Postal 4-116, Itzimná, 97000 Mérida, Yucatán Mexico

**Correction: Planta (2023) 257:42** 10.1007/s00425-023-04075-6

The article Effect of herbivore load on VOC‑mediated plant communication in potato, written by Carla Vázquez‑González, Violeta Quiroga, Lucía Martín‑Cacheda, Sergio Rasmann, Gregory Röder, Luis Abdala‑Roberts, Xoaquín Moreira, was originally published Online First without Open Access. After publication in volume 257, issue 2, pages 1–6, CRUE-CSIC consortium requested that the article be Open Choice to make the article an open access publication. Open access funding provided by Misión Biológica de Galicia (MBG-CSIC), within the CRUE-CSIC Agreement. Therefore, the copyright of the article has been changed to © The Author(s) 2024 and the article is forthwith distributed under the terms of the “Creative Commons Attribution 4.0 International License, which permits use, sharing, adaptation, distribution and reproduction in any medium or format, as long as you give appropriate credit to the original author(s) and the source, provide a link to the Creative Commons licence, and indicate if changes were made. The images or other third party material in this article are included in the article’s Creative Commons licence, unless indicated otherwise in a credit line to the material. If material is not included in the article’s Creative Commons licence and your intended use is not permitted by statutory regulation or exceeds the permitted use, you will need to obtain permission directly from the copyright holder. To view a copy of this licence, visit http://creativecommons.org/licenses/by/4.0/.”

The original article has been corrected.

