# Supplementary material for: Effect of herbivore load on VOC-mediated plant communication in potato
Source: Planta. 2023 Jan 23;257(2):42. doi: 10.1007/s00425-023-04075-6 (PMC11568002; doi:10.1007/s00425-023-04075-6)
Supplement: Supplementary file 1 — Supplementary file1 (DOCX 21 KB) [file 425_2023_4075_MOESM1_ESM.docx]

**Effect of herbivore load on VOC-mediated plant-plant signalling in potato**

Carla Vázquez-González, Violeta Quiroga, Lucia Martín-Cacheda, Sergio Rasmann, Gregory Röder, Luis Abdala-Roberts and Xoaquín Moreira

**SUPPLEMENTARY MATERIALS**

**Table S1** Means (± SE) for emission of individual volatile organic compounds (ng·h^-1^) identified by GC-MS (see below) for each level of the herbivory treatment with *Spodoptera exigua* (control vs. low herbivore load vs. high herbivore load) in potato (*Solanum tuberosum*) emitter plants. For VOC collection we bagged plants with a 2-L Nalophan bag and trapped VOCs on a charcoal filter (SKC sorbent tube filled with anascorb CSC coconut-shell charcoal) for two hours at a rate of 0.25 L min^-1^. We eluted traps with 150 μL dichloromethane (CAS#75-09-2, Merck, Dietikon, Switzerland) to which we had previously added one internal standard (tetralin 200 ng in 10 μL dichloromethane). We then injected 1.5 μL of the extract for each sample into an Agilent 7890B gas chromatograph (GC) coupled with a 5977B mass selective detector (MSD) fitted with a 30 m × 0.25 mm × 0.25 μm film thickness HP-5MS fused silica column (Agilent, Santa Clara, CA, USA). We operated the GC injector in pulsed splitless mode (250 ºC, injection pressure 15 psi) with helium as the carrier gas. The GC oven temperature programme was: 3.5 min hold at 40ºC, 5ºC min^-1^ ramp to 230ºC, then a 3 min hold at 250ºC post run (constant helium flow rate 0.9 mL min^-1^). Transfer line was set at 280 ºC. In the MS detector (EI mode), a 33-350 (m/z) mass scan range was used with MS source and quadrupole set at 230ºC and 150ºC, respectively. RT = Retention times. KRI = Kovats retention index used for identification of compounds without commercial standards (KRIc for calculated values and KRIe for expected values from the NIST database).

| **Compound** | **RT** | **KRIc** | **KRIe** | **Control** | **Low Herbivore**  **load** | **High Herbivore**  **load** |
| --- | --- | --- | --- | --- | --- | --- |
| α-Pinene† | 9.605 |  |  | **1.24 ± 0.25 (a)** | **1.19 ± 0.35 (b)** | **0.4 ± 0.16 35 (b)** |
| 3-Hexen-1-ol, acetate, (E)- | 12.072 | 1006.97 | 1006 | 0.09 ± 0.09 | 0.99 ± 0.48 | 1.02 ± 0.6 |
| D-Limonene | 12.763 | 1028.56 | 1030 | **0.09 ± 0.09 (a)** | **0.41 ± 0.14 (ab)** | **1.61 ± 0.91 35 (b)** |
| Linalool | 15.008 | 1098.72 | 1099 | **0.57 ± 0.21 (a)** | **2.24 ± 0.4 (b)** | **2.6 ± 0.61 35 (b)** |
| Nonanal | 15.171 | 1103.97 | 1104 | 2.33 ± 0.48 | 2.3 ± 0.31 | 2.67 ± 0.56 |
| Nonatriene | 15.566 | 1116.82 | 1116 | **9.16 ± 4.23 (a)** | **34.7 ± 12.64 (b)** | **34.66 ± 11.8 35 (b)** |
| Butanoic acid, 3-hexenyl ester, (E) | 17.686 | 1185.78 | 1185 | **0 ± 0 a** | **1.55 ± 0.52 (b)** | **4.25 ± 0.82 (c)** |
| Dodecane† | 18.061 |  |  | 4.86 ± 0.54 | 4.52 ± 0.33 | 6.06 ± 1.42 |
| Decanal | 18.29 | 1205.91 | 1206 | 0.36 ± 0.22 | 0.61 ± 0.23 | 0.8 ± 0.26 |
| (-)-Bornyl acetate | 20.516 | 1284.65 | 1285 | 0.47 ± 0.19 | 0.52 ± 0.19 | 0.32 ± 0.2 |
| Tridecane† | 20.928 |  |  | 2.76 ± 0.46 | 2.55 ± 0.28 | 3.74 ± 1.13 |
| α-Copaene | 23.062 | 1377.36 | 1378 | **1.65 ± 0.76 (a)** | **4.71 ± 1.66 (b)** | **3.26 ± 0.71 35 (b)** |
| β-Elemene | 23.466 | 1392.16 | 1391 | **0.77 ± 0.42 (a)** | **2.08 ± 0.88 (ab)** | **3.66 ± 1.89 35 (b)** |
| (+)-Sativene | 23.604 | 1397.21 | 1396 | 2.75 ± 0.51 | 2.5 ± 0.36 | 3.59 ± 0.66 |
| α-Gurjunene† | 23.976 |  |  | 3.81 ± 1.63 | 2.31 ± 0.74 | 5.64 ± 2.49 |
| β-Caryophyllene† | 24.261 |  |  | **39.31 ± 14.65 (a)** | **32.1 ± 5.44 (ab)** | **68.64 ± 30.49 35 (b)** |
| trans-α-Bergamotene | 24.664 | 1438.83 | 1440 | 0.49 ± 0.3 | 0.61 ± 0.2 | 1.15 ± 0.52 |
| cis-β-Farnesene | 24.804 | 1444.36 | 1444 | 3.21 ± 1.34 | 1.84 ± 0.52 | 3.67 ± 1.21 |
| 5,9-Undecadien-2-one, 6,10-dimethyl-, (E)† | 25.008 |  |  | **0.68 ± 0.24 (a)** | **1.34 ± 0.34 (ab)** | **2.44 ± 0.66 35 (b)** |
| (E)-β-Farnesene† | 25.117 |  |  | 6.3 ± 3.13 | 5.36 ± 1.59 | 12.67 ± 6.03 |
| Patchoulene | 25.396 | 1467.72 | 1467 | 2.16 ± 1.06 | 1.69 ± 0.73 | 2.62 ± 1.43 |
| Acoradien | 25.46 | 1470.25 | 1471 | 2.29 ± 0.42 | 2.66 ± 0.62 | 3.26 ± 0.68 |
| γ-Selinene | 25.678 | 1478.85 | 1479 | 0.46 ± 0.23 | 1.3 ± 0.62 | 2.79 ± 1.16 |
| Germacrene D | 25.789 | 1483.21 | 1481 | 1.6 ± 1.01 | 1.3 ± 0.41 | 4.36 ± 2.47 |
| β-Selinene | 25.913 | 1488.12 | 1486 | **2.02 ± 0.89 (a)** | **10.87 ± 4.92 (b)** | **14.97 ± 6.1 35 (b)** |
| Zingiberene | 26.078 | 1494.63 | 1495 | 1.6 ± 0.29 | 3.28 ± 1.04 | 4.19 ± 1.12 |
| α-Farnesene | 26.39 | 1507.29 | 1508 | **0.31 ± 0.15 (a)** | **2.01 ± 0.77 (b)** | **3.66 ± 1.24 35 (b)** |
| β-Sesquiphellandrene | 26.794 | 1524.04 | 1524 | **2.58 ± 1.22 (a)** | **2.52 ± 0.84 (ab)** | **5.01 ± 1.97 35 (b)** |
| unidentified sesquiterpenoid | 28.114 | 1578.94 |  | 4.91 ± 2.21 | 15.16 ± 7.14 | 8.57 ± 3.94 |
| Caryophyllene oxide† | 28.295 |  |  | 1.73 ± 0.64 | 0.87 ± 0.27 | 1.73 ± 0.65 |
| Viridiflorol | 28.843 | 1609.53 | 1611 | 0.38 ± 0.22 | 0.7 ± 0.3 | 1.17 ± 0.52 |

^1^We performed *P*-value adjustments using the False Discovery Rate for *P* < 0.05 to avoid inflating Type I error due to multiple testing. Individual VOCs that significantly differed among treatments (*P* < 0.05) are in bold. Letters in parenthesis.

†Compounds identified with pure commercial standards.
